# Cross-Neutralization of Distant Coronaviruses Strongly Correlates with Spike S2-Specific Antibodies from Immunocompetent and Immunocompromised Vaccinated SARS-CoV-2-Infected Patients

**DOI:** 10.3390/vaccines13090949

**Published:** 2025-09-04

**Authors:** Sara V. Patel, Brooke M. Leeman, Patricia J. Botros, Joanna Folta, Dhiman Shahid, Anya I. Rocque, Andrew S. Joyal, Joseph A. Vecchio, Eliza Passell, Dessie Tien, Zahra Reynolds, Karry Su, Tammy D. Vyas, Jatin M. Vyas, Emory Abar, Mamadou Barry, Andrew Alexandrescu, Zachary Wallace, Jeffrey M. DaCosta, Manish C. Choudhary, Trevor J. Tamura, Gregory E. Edelstein, Yijia Li, Rinki Deo, Jeffrey A. Sparks, Julie Boucau, Owen T. Glover, Amy K. Barczak, Jacob Lemieux, Mark J. Siedner, Jonathan Z. Li, Ismael Ben Fofana

**Affiliations:** 1 Biology Department, Boston College, 140 Commonwealth Avenue, Chestnut Hill, MA 02467, USA; sarapatel5@yahoo.com (S.V.P.); jvecchi2@nd.edu (J.A.V.); jeffrey.dacosta@bc.edu (J.M.D.); 2 Massachusetts General Hospital, Harvard Medical School, Cambridge, MA 02114, USA; 3 Brigham and Women’s Hospital, Harvard Medical School, Boston, MA 02115, USAjsparks@bwh.harvard.edu (J.A.S.);; 4 Ragon Institute of MGH, MIT, and Harvard, Boston, MA 02139, USAoglover@mgh.harvard.edu (O.T.G.);

**Keywords:** SARS-CoV-2, COVID-19, neutralizing antibodies, S2-specific antibodies, spike-S1 dominance, immunocompromised

## Abstract

**Background/Objectives**: Despite the lifting of the COVID-19 public health emergency, SARS-CoV-2 infections continue to be recorded worldwide. The continued prevalence of infection has been attributed to the ability of the virus to evade host immune responses, including neutralizing antibody-derived immunity. The vast majority of antibody escape mutations has been associated with the S1 subunit of the spike protein. The other region of the spike, the S2 subunit, is the most conserved region amongst coronaviruses. We hypothesized that S2-specific antibody levels are modest in vaccinated and SARS-CoV-2-infected patients, resulting in suboptimal neutralization of distant coronaviruses. **Methods**: Here, we analyzed S1- and S2-specific antibody levels in SARS-CoV-2-infected individuals, including a mixed cohort of those with and without immunosuppression and prior vaccination. **Results**: We found that S2-specific antibody responses were generally lower than S1-specific antibody responses. Intriguingly, Omicron-S1-specific antibody levels were higher than Wuhan-S1-specific antibody levels despite all vaccinated participants having received Wuhan-spike-based immunogens. This emphasizes the importance of the infecting variant and vaccine immunogen in the production of spike-targeting antibodies and associated hybrid immunity. Although S1-specific antibody levels were generally higher than their S2-specific counterparts, the correlation between neutralization and binding antibody levels was mostly higher in S2- compared with S1-specific responses. **Conclusions**: We conclude that S2-based immunogens are suitable for the induction of antibody-based immunity against novel SARS-CoV-2 variants but also against more distant coronaviruses, which would support a better protection for the immunocompromised as well as other vulnerable populations.

## 1. Introduction

Coronavirus disease of 2019 (COVID-19) emerged in late 2019 in Wuhan (China) [1] and rapidly expanded across the globe; the World Health Organization (WHO) declared a global health pandemic in March 2020 [2]. A number of vaccine candidates were developed, including by Pfizer-BioNTech and Moderna, with emergency use authorization granted in the United States (US) within a year [3]. Millions of people were immunized worldwide, and many lives were saved [4]. In the US, the COVID-19 public health emergency expired in May 2023 [5]. However, the virus has continued to evolve, with variants such as Delta and Omicron emerging around the globe [6]. This evolution of the virus raises a concern regarding the longevity of vaccine-induced protection among the most vulnerable sections of the population, especially immunocompromised individuals [7,8,9,10].

The titer of neutralizing antibodies against SARS-CoV-2 has been identified as a predictor of COVID-19 disease severity [11]. COVID-19 disease severity has also been associated with high levels of SARS-CoV-2 antibodies by other studies, which did not always distinguish between binding antibody titers and neutralization potency [12,13]. SARS-CoV-2 was also shown to evade antibody responses through S1-region mutations, including the receptor binding domain [11,14,15,16,17]. Notably, the ability of SARS-CoV-2 to evade antibody neutralization is not limited to the receptor binding domain (RBD) [18]. Nucleotide deletions were observed in the N-terminal Domain (NTD) of the S1 subunit of the spike protein, leading to escape from neutralizing antibody responses [18]. The ability of SARS-CoV-2 to escape antibody responses, together with the well-established importance of neutralization antibody responses to disease progression and survival, further underscores the importance of understanding vaccine protection among immunocompromised individuals [7,8,9,10].

Here, we measured the levels of antibody responses targeting the S1 and S2 regions of the spike protein from serum of vaccinated individuals with non-severe COVID-19, including those with and without immunosuppression. We then analyzed correlations between neutralization of wild-type Wuhan, Omicron BA.1, SARS-CoV, and WIV1-CoV pseudoviruses. We found that S1-specific antibody responses were more abundant while S2-specific antibody levels were lower and that both were insufficient for potent neutralization of distant coronaviruses, SARS-CoV and WIV1-CoV. Furthermore, we found that S1- and S2-specific antibody levels were generally correlated with pseudovirus neutralization for the immunocompetent, with a mixed result for the immunocompromised participants. Even though S1-specific antibody levels were generally higher compared with their S2-specific counterparts, we found correlations between S2-specific antibody responses and virus neutralization to be greater than those observed with S1-specific antibodies.

## 2. Methods

### 2.1. Study Enrollment and Sample Collection

Serum samples were obtained from the prospective Post-Vaccination Viral Characteristics Study (POSITIVES) between January 2021 and May 2023. POSITIVES is a collaborative study at Mass General and Brigham between the laboratories of Dr. Amy K. Barczak (the Ragon Institute of Massachusetts General Brigham, MIT, and Harvard, Boston, Massachusetts), Dr. Jacob E. Lemieux (Massachusetts General Hospital, the Broad Institute, MIT, Harvard Medical School, and Tufts Medical School), Dr. Jonathan Z. Li (Brigham and Women’s Hospital and Harvard Medical School); and Dr Mark J. Siedner (Massachusetts General Hospital and Harvard Medical School). The principal aim of the study is to understand the duration and determinants of viral shedding with acute COVID-19 infection.

The study has previously been described in full detail [10,19] In brief, study staff use automated lists of individuals in the Mass General Brigham medical record system with confirmed COVID-19 infection to recruit potential participants. The study population is demographically similar to the geographic region [20]. Eligibility criteria include the presence of a recent (within 5 days) positive test for COVID-19 infection, age 18 or older, and primary residence in the hospital catchment area (to enable shipment and remote collection of study specimens). Consenting individuals are sent nasal swabs and asked to self-sample their nares every other day for 14 days and weekly thereafter until they have two consecutive negative samples by PCR. Individuals also provide blood for immunologic profiling at enrollment and at days 14, 180, and 360 after enrollment.

Clinical study staff conduct a chart review for each participant to determine the presence and nature of immunosuppression, which included seventeen immunocompromised participants (n = 17) categorized into non-severely (n = 11) and severely immunocompromised individuals (n = 6), as previously described [10]. The severely immunocompromised were individuals with severe hematological malignancy/transplant patients (S-HT) and patients with severe autoimmune disorders (S-A, participants with autoimmune condition receiving B-cell targeting agents or B cell deficiency) as previously categorized and non-severe immunodeficiency (NS) [21,22].

### 2.2. Ethics Declaration

This study was approved by the Institutional Review Boards of Boston College (IRB Protocol Number # 21.115.01e) and Mass General Brigham (IRB# 2021P000812). Informed consent was obtained from all participants. The authors confirm that all research was performed in accordance with relevant guidelines/regulations.

### 2.3. Cell Lines

The 2 cell lines used in this study, Human Kidney Embryonic cells (HEK293T) and HEK293T cells engineered to express the Angiotensin Convertase Enzyme 2 (293T-ACE2), have previously been described [23,24]. HEK293T-ACE2 cells were a gift from Dr. Huihui Mou and Dr. Michael Farzan (SCRIPPS Research Institute, FL, USA).

### 2.4. SARS-CoV-2-Specific Antibody Measurement by ELISA

#### 2.4.1. ELISA Antigens

Soluble SARS-CoV-2 spike S1 and S2 corresponding to the Wuhan and Omicron (B.1.1.529) variants were obtained from Acro Biosystems (Newark, DE, USA). Wuhan version of SARS-CoV-2 spike S1 protein, His Tag (Acro Biosystems catalog # S1N-C52H3), contains AA Val 16-Arg 685 (Accession # QHD43416.1). Wuhan version of SARS-CoV-2 spike S2 protein, His Tag (Acro Biosystems catalog # S2N-C52H5), contains AA Ser 686–Pro 1213 16-Arg 685 (Accession # QHD43416.1). Omicron/BA.1 version of SARS-CoV-2 Spike S2, His Tag (Acro Biosystems catalog # S2N-C52Hf), contains AA Ser 686-Pro 1213 (Accession # QHD43416.1 (N764K, D796Y, N856K, Q954H, N969K, L981F, F817P, A892P, A899P, A942P, K986P, V987P). Mutations were identified on the SARS-CoV-2 Omicron variant (Pango lineage: BA.1; GISAID clade: GRA; Nextstrain clade: 21K). SARS-CoV-2 spike S1, His Tag (B.1.1.529/Omicron) (S1N-C52Ha), contains AA Val 16-Arg 685 (Accession # QHD43416.1 (A67V, HV69-70del, T95I, G142D, VYY143-145del, N211del, L212I, ins214EPE, G339D, S371L, S373P, S375F, K417N, N440K, G446S, S477N, T478K, E484A, Q493R, G496S, Q498R, N501Y, Y505H, T547K, D614G, H655Y, N679K, P681H)). The spike mutations were identified on the SARS-CoV-2 Omicron variant (Pango lineage: B.1.1.529; GISAID clade: GR/484A; Nextstrain clade: 21K).

#### 2.4.2. S2 Antigen Modification for Solubility

Proline substitutions (F817P, A892P, A899P, A942P, K986P, V987P) were introduced in both spike S2 (Wuhan and Omicron versions) by the manufacturer (Acro Biosystems) in order to prevent the formation of aggregates in the course of protein production.

#### 2.4.3. ELISA Procedure

ELISA was performed as previously described [11,23,25]. Briefly, 96-well Nunc MaxiSorp ELISA plates (Thermo Scientific, Waltham, MA, USA) were coated with viral antigens (Wuhan S1, Wuhan S2, Omicron S1 or Omicron S2) diluted in carbonate–bicarbonate buffer to a concentration of 1 µg/mL before incubation for 1 h at room temperature. Plates were washed with a buffer consisting of 50 mM Tris (pH 8.0) (ThermoFisher), 140 mM NaCl (MilliporeSigma, Burlington, MA, USA), and 0.05% Tween-20 (ThermoFisher). Next, plates were incubated with a blocking buffer consisting of 1% BSA (MilliporeSigma), 50 mM Tris (pH 8.0), and 140 mM NaCl for 30 min at room temperature. The plates were washed 1–3 times after blocking. Serum samples were diluted 1:100 with a dilution buffer consisting of 1% BSA, 50 mM Tris (pH 8.0), 140 mM NaCl, and 0.05% Tween-20. After sample addition, plates were incubated at 37 °C for 30 min followed by washing, 5 times. Serum IgG levels were detected by addition of HRP-conjugated anti-human-IgG purchased from ThermoFisher (catalog # 62–8420) and diluted (1:4000). The plates were incubated for 30 min at room temperature. After the washes, TMB substrate (ThermoFisher) was added to each plate for 10 min, and the reaction was terminated with TMB stop solution (Southern Biotech, Birmingham, AL, USA). Data were acquired by spectrophotometry at 450 nm using a Victor X5 microplate reader (Perkin Elmer, Shelton, CT, USA).

### 2.5. SARS-CoV-2 Pseudovirus Production

Pseudovirus production and titration processes have previously been described [11,26,27]. The plasmids obtained from Addgene (Watertown, MA, USA) were gifted by Dr. Alejandro Balazs. A group of 4 plasmids—pHAGE-CMV-luc2-IRES-ZsG-W (Addgene plasmid # 164432), pRC-CMV-Rev1b (Addgene plasmid # 164443), pHDM-Tat1b (Addgene plasmid # 164442), and pHDM-Hgpm2 (Addgene plasmid # 164441)—were used for production of all pseudovirus variants. Only the plasmid corresponding to the spike differed for the 4 viruses. The plasmids pTwist-SARS-CoV-2 ∆18 (Addgene plasmid # 164436), pTwist-SARS-CoV-2 ∆18 B.1.1.529 (Addgene plasmid # 1789907), pTwist-WIV1-CoV ∆18 (Addgene plasmid # 164439), and pTwist-SARS-CoV ∆18 (Addgene plasmid # 169465) were used for production of the Wuhan, Omicron, SARS-CoV, and WIV1-CoV pseudoviruses, respectively. A total of 5 plasmids were therefore used for each of the 4 pseudoviruses, with the spike expression plasmid being the only variable. On the day before transfection, 12–15 million HEK293 T cells were seeded in T175 (ThermoFisher) in presence of 25 mL of Dulbecco’s modified Eagle’s medium (DMEM) supplemented with fetal bovine serum (FBS) at 10% of the total volume (DMEM10) (ThermoFisher) and containing penicillin/streptomycin (ThermoFisher). Before transfection, culture media was replaced with fresh 25 mL of DMEM10. The transfection was performed with GenJet (SignaGen Laboratories, Frederick, MD, USA) according to the manufacturer’s recommendations. Twenty-four hours later, transfection media was replaced with fresh DMEM10, and culture supernatant containing secreted pseudoviruses was harvested 5 days post-transfection and cleared using a 0.45 µm Nalgene syringe filter (ThermoFisher). The pseudovirus preparation was divided into 1 mL aliquots per cryovial and stored at −80 °C.

#### 2.5.1. SARS-CoV-2 Pseudovirus Titration

Titration of pseudovirus preparations has been previously described [11,23,25]. Here, 293T-ACE2 cells (10^4^ cells/well) were seeded in 100 µL of DMEM10 into 96-well black/clear bottom plates purchased from ThermoFisher (catalog # 165305). For titration, 50 µL of 2x serially diluted pseudovirus preparation was added to corresponding wells. Control (background) wells received 50 µL of DMEM10. On the fifth day, pseudovirus infectivity was quantified by luciferase assay using the previously described in-house luciferin buffer [11,26]. Assay plates were read using a Victor X5 microplate reader (Perkin Elmer).

#### 2.5.2. SARS-CoV-2 Pseudovirus Neutralization Assay

Pseudovirus neutralization has previously been described [11,23,25]. All reagents, cells, viruses, and sera were added in a single streamline with incubation and assay readout in the same plate, ThermoFisher 96-well black/clear bottom plates. A luciferase readout of 30,000 luminescence rate units (LRU) was targeted as viral input with a 5-day incubation period. Patients’ sera were diluted with DMEM10 starting at 10-fold dilution and performing 3-time serial dilutions (from 1/10 to 1/21,870). A starting dilution of 20× (1/20 to 1/43,740) and 30× (1/30 to 1/65,610), when necessary, was applied to the samples for which the 10-fold dilutions were insufficient to cross the 50% neutralization mark. Fifty µL of pseudovirus preparations were added onto the diluted sera, and the mixtures were incubated for 1 h at 37 °C before addition of HEK293T-ACE2 cells (10^4^ cells/well) prepared in 50 µL of DMEM10. Background wells containing cells only were prepared, while cells plus virus only (no sera) were prepared as positive controls corresponding to 100% assay readout. The plates were incubated at 37 °C, 5% CO_2_, and 70% humidity for 5 days. Following transduction, cells were lysed and luciferase assay performed as previously described [11,23,26]. Fifty microliters of luciferin buffer containing 20 mM Tris-HCl (ThermoFisher), 100 mM EDTA (ThermoFisher), 1 mM MgCl_2_ (ThermoFisher), 26.5 mM MgSO_4_ (ThermoFisher), 17 mM dithiothreitol (Goldbio, St. Louis, MO, USA), 250 mM Adenosine-5′-Triphosphate (Goldbio), and 750 mM D-luciferin (Goldbio) was added to the well and incubated for 5 min with agitation before luminescence was quantified within 30 min of buffer addition using a Victor X5 microplate reader (Perkin Elmer). Neutralization curves were analyzed using GraphPad prism. Neutralizing antibody responses (NT50) were calculated by taking the inverse of the 50% inhibitory concentration value for each sample. Of note, the inverse serial dilution number was multiplied by two to obtain the final NT50 values because (diluted) sera were further diluted with equal volumes of pseudovirus during the serum–virus incubation step.

### 2.6. Statistical Analysis

Graphpad Prism 9 (v9.3.1) was used to analyze neutralization data and determine the 50% neutralization titer (NT50). R (v4.2.1) was used for all other statistical analyses. Antibody binding means were analyzed using *t*-tests when comparing two groups (e.g., S1- vs. S2-specific binding), with a paired test when the same patients were sampled in both groups. Neutralizing antibody titer means were compared using a one-way ANOVA when all samples were independent, and linear mixed models with patient ID as a random effect when the same participant was measured for multiple antigens/pseudoviruses. Due to considerable skew in NT50 values, in these analyses, these values were transformed (log10 NT50+1). For significant ANOVA and linear mixed models, differences among groups were identified with the post hoc Tukey HSD test. Correlations between antibody binding and neutralization (using log10 NT50+1 transformation) were analyzed using the nonparametric Spearman’s rank correlation test, with corrections for multiple comparisons using the Benjamini–Hochberg method. Alpha of 0.05 was used to determine statistical significance in all tests.

## 3. Results

**Figure 1:**
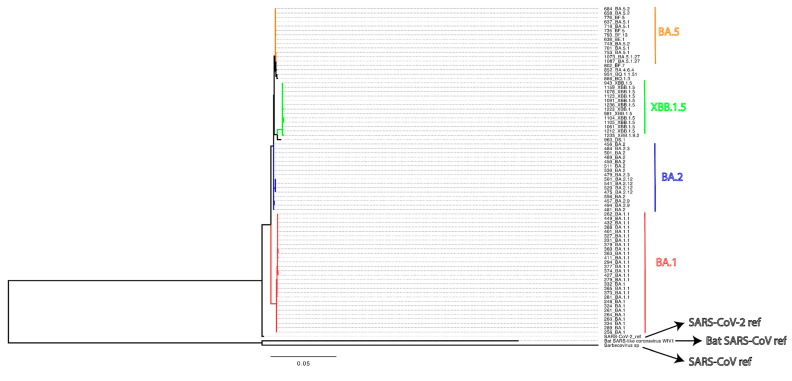
Maximum likelihood phylogenetic tree based on SARS-CoV-2 spike gene sequences, rooted to the SARS-CoV-2 (Wuhan-Hu-1 reference genome) with the addition of SARS-CoV reference genome and Bat SARS-CoV reference genome (WIV1-CoV).

The samples from the POSITIVES study cohort were collected between 14 days and 48 days from the first PCR test (median: 20 days; interquartile range: 7 days; 75% of samples were collected less than 24 days post-first test). SARS-CoV-2 variant information from the current study are presented in a phylogenetic tree with Wuhan, SARS-CoV, and WIV1-CoV as references (Figure 1). Eight of the eighty-seven participants were non-vaccinated, and one participant had received a single dose of the Johnson & Johnson COVID-19 vaccine (Supplementary Table S1). All other participants (n = 78) had received between two and five doses of either the Moderna and/or Pfizer/BioNTech COVID-19 mRNA vaccines. All three vaccines were based on a Wuhan spike trimer formulation at the time of this analysis. Table 1 presents the demographic and clinical characteristics of the participants. Supplementary Table S1 presents information on the number of participants, viral variants, vaccine doses, and timing, which was obtained from their medical records.

The tree was constructed using IQ-TREE under the HKY+F+I nucleotide substitution model, selected as the best fit, with 1000 ultrafast bootstrap replicates to assess branch support. The analysis included all 75 available sequences. Key SARS-CoV-2 lineages, including BA.1 (referred to here as Omicron), BA.2, BA.5, and XBB.1.5, are annotated. The scale bar represents the number of nucleotide substitutions per site.

### 3.1. Validity of Modified Soluble S2 as ELISA Antigens

We first sought to measure antibody levels against soluble S1 and S2 antigens. The amino acid sequences that corresponded to the wild-type Wuhan and the Omicron B.1.1.529 variant (also known as the Omicron BA.1 variant and hereafter referred to as Omicron) were compared to the soluble Wuhan S2 and Omicron S2, which had been mutated for solubility by the manufacturer (Acro Biosystems). The analysis also included the S1 and S2 subunits corresponding to SARS coronavirus (SARS-CoV) and the bat SARS-like coronavirus WIV1 (WIV1-CoV).

The soluble Wuhan S2 and Omicron S2 antigens were similar and clustered to their corresponding wild-type Wuhan S2 and Omicron S2 subunits (Supplementary Figure S1). In general, comparison of S1 and S2 subunits confirmed the similarities between Wuhan and Omicron and their isolation from the distant relatives SARS-CoV and WIV1-CoV (Supplementary Tables S2–S4).

### 3.2. S1-Specific Antibody Levels Surpassed S2-Specific Antibody Levels

**Figure 2:**
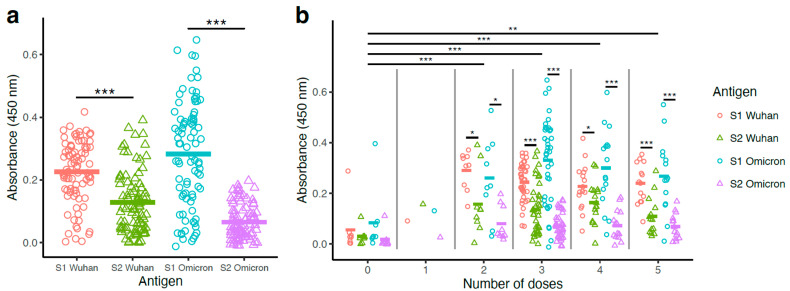
High S1-specific and low S2-specific antibody levels were observed with vaccinated and SARS-CoV-2-infected individuals. Binding antibody levels were measured using serum obtained from 87 study participants. Serum binding antibody titers were measured by ELISA using S1-Wuhan, S2-Wuhan, S1-Omicron, and S2-Omicron as antigens. Antibodies were detected using HRP conjugated secondary anti-human IgG. Absorbance was determined at 450 nm. (**a**) S1- and S2-specific antibody levels from all participants are shown. Paired *t*-tests were used to compare antibody absorbance for S1 vs. S2 regions of Wuhan and Omicron antigens. (**b**) S1- and S2-specific antibody levels from all participants are shown according to the number of vaccine doses. For each dose number, paired *t*-tests were used to compare antibody absorbance for S1 vs. S2 regions of Wuhan and Omicron antigens. Means for each number of doses were compared using a linear mixed model with individual patient identification as a random effect. Statistical significance was defined as * *p* < 0.05, ** *p* < 0.01, and *** *p* < 0.001.

Antibody levels were measured by ELISA using the S1 and S2 antigens from the Wuhan and Omicron strains in the form of soluble proteins (Figure 2). S1-Wuhan-specific antibody levels (mean = 0.23) were significantly higher than S2-Wuhan-specific antibody levels (mean = 0.13) (paired *t*-test; *p* < 0.001) (Figure 2a). The same pattern was seen for Omicron, with the S1-Omicron-specific antibody levels (mean = 0.28) significantly higher than S2-Omicron-specific antibody levels (mean = 0.07) (paired *t*-test; *p* < 0.001) (Figure 2a). Overall, S1-Omicron-specific antibody levels were the highest, while S2-Omicron-specific antibody levels were the lowest (Figure 2a). Although vaccinated participants received mRNA vaccine corresponding to the Wuhan spike, Wuhan-S1-specific antibody levels were unexpectedly lower than Omicron-S1-specific antibody levels (Figure 2a). On the contrary, Wuhan-S2-specific antibody levels were higher than Omicron-S2-specific antibody levels (Figure 2a).

### 3.3. Binding Antibody Titers Were Higher with Vaccinated Groups Compared to the Non-Vaccinated Group, with No Significant Differences Among the Booster Groups (2–5 Doses)

Samples were subsequently analyzed according to the vaccination status and number of doses for comparison of S1- and S2-specific antibody levels. Vaccinated participants (1–5 doses; *n* = 79) presented higher antibody titers than non-vaccinated ones (0 doses; *n* = 8) (Figure 2b).

The one-dose vaccination group had only one participant, which reduced the power of statistical analyses involving this group. The remaining vaccination groups (doses 2–5) had significantly higher antigen binding compared with non-vaccinated participants (linear mixed model with Tukey HSD post hoc test; *p* < 0.01). However, no significant differences in antibody levels were observed among the multiple booster groups (two to five doses; Figure 2b).

Inter-group analyses were followed up with intra-group analyses, in which paired *t*-tests were used to assess if participants had higher antibody levels for S1- or S2-specific antibodies. For groups with either zero or one dose, no significant differences were found, possibly due to the low sample size and reduced statistical power (Figure 2b). For groups with 2–5 doses, all tests found significantly higher antibody levels with S1 compared with S2-specific antigens. For example, in participants who received three doses (n = 39), S1-Wuhan antibody levels (mean = 0.24) were significantly higher than S2-Wuhan levels (mean = 0.13) (paired *t*-test; *p* < 0.001), and S1-Omicron antibody levels (mean = 0.33) were significantly higher than S2-Omicron levels (mean = 0.07) (paired *t*-test; *p* < 0.001) (Figure 2b).

### 3.4. Pseudovirus Neutralization Was Higher with Wuhan and Omicron Compared to SARS-CoV and WIV1-CoV

Sera from all participants (n = 87) were evaluated for neutralization capacity of several coronaviruses. We used a previously published pseudovirus-based neutralization assay [11,14,23] with pseudoviruses corresponding to the Wuhan and Omicron variants as well as the distant relatives SARS-CoV and WIV1-CoV [28]. Neutralizing antibody responses (NT50) were calculated by taking the inverse of the 50% inhibitory concentration value for each sample (Supplementary Figure S2).

**Figure 3:**
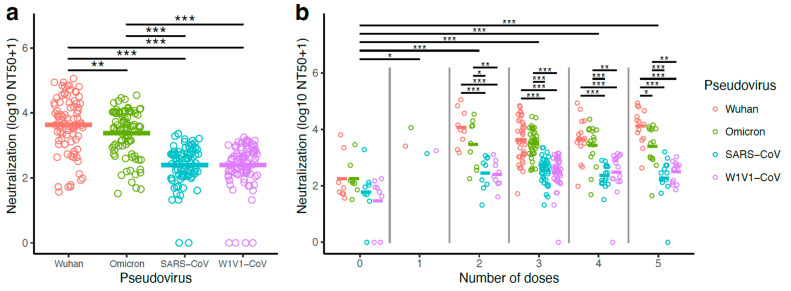
Highest antibody neutralization concentrations were observed against Wuhan and Omicron pseudoviruses. Neutralizing antibody levels were measured using serum obtained from 87 study participants. Neutralization concentrations were reported as a 50% neutralization titer (NT50). Values were transformed due to a skew in the data (log10 of NT50+1 transformation). (**a**) Neutralization titers for Wuhan, Omicron, and SARS-CoV and WIV1-CoV pseudoviruses are shown. Titer means of pseudoviruses were compared using a linear mixed model with individual patient identification as a random effect. (**b**) Neutralization titers analyzed according to the number of vaccine doses are shown. Similar linear mixed models were used to compare values within and among the number of doses. Statistical significance was defined as * *p* < 0.05, ** *p* < 0.01, and *** *p* < 0.001.

Highest serum antibody neutralization concentrations were observed with Wuhan pseudovirus (Figure 3). Nine of the samples required a higher serum starting dilution of 20×, and three samples required a higher serum starting dilution of 30× for the crossing of the 50% neutralization levels in order to generate the NT50 values (Supplementary Figure S3). Overall, strong serum antibody neutralization concentrations were observed against the Omicron pseudovirus, but these were significantly lower when compared to the Wuhan pseudovirus neutralization (log_10_ NT50+1 transformation; Wuhan mean: 3.63, Omicron B1.1.259 mean: 3.37; linear mixed model with Tukey HSD post hoc test; *p* < 0.01, Figure 3a).

Lower serum neutralization concentrations were observed with the more distantly related SARS-CoV (log_10_ NT50+1 transformation; mean = 2.40) and WIV1-CoV (mean = 2.39) pseudoviruses. Wuhan pseudovirus neutralization concentrations were significantly higher than those observed with both SARS-CoV (linear mixed model with Tukey HSD post hoc test; *p* < 0.001) and WIV1-CoV (linear mixed model; *p* < 0.001) (Figure 3a). Similar results were recovered when comparing Omicron pseudovirus neutralization concentrations to those with SARS-CoV and WIV1-CoV (linear mixed model with Tukey HSD post hoc test; *p* < 0.001; Figure 3a). No significant differences were observed between SARS-CoV and WIV1-CoV pseudovirus neutralization (*p* > 0.05; Figure 3a).

### 3.5. Vaccination Significantly Improved Pseudovirus Neutralization Potency

Vaccinated participants (1–5 doses; n = 79) presented higher neutralization concentrations, compared with non-vaccinated participants (0 doses; n = 8) (Figure 3b). Neutralization was lowest in the zero-dose group (log10 NT50+1 transformation; mean = 1.94), and the means of all other dose groups were significantly higher. This was found even for the comparison to the one-dose group that included only one participant (mean = 3.47; linear mixed model with Tukey HSD test; *p* < 0.05). Neutralization was further elevated in participants who had had boosters (2-dose mean = 3.10; 3-dose mean = 3.05; 4-dose mean = 2.99; 5-dose mean = 3.08). Comparisons of the non-vaccinated (0-dose) to these groups with boosters (2–5 dose groups) were all highly significant (linear mixed model with Tukey HSD test; *p* < 0.001; Figure 3b). Intra-group analyses (i.e., within those with the same number of vaccine doses) did not find differences among pseudovirus neutralization for those within the zero-dose and one-dose groups. Within groups of participants with multiple doses (2–5 doses), pseudovirus neutralization was significantly higher for Wuhan and Omicron, compared with SARS-CoV and WIV1-CoV (linear mixed model with Tukey HSD test; all *p* < 0.05; Figure 3b).

### 3.6. S1- and S2-Specific Antibody Titers Generally Correlated Positively with Pseudovirus Neutralization Potency

We next evaluated the relationship between antigen-specific antibody titers and neutralization capacity. Linear correlations between pseudovirus neutralization concentrations and S1- or S2-specific Wuhan/Omicron antibody levels were analyzed for all participants (n = 87). Separate analyses were performed for each pseudovirus (Wuhan, Omicron, SARS-CoV, and WIV1-CoV), resulting in 16 comparisons.

**Figure 4:**
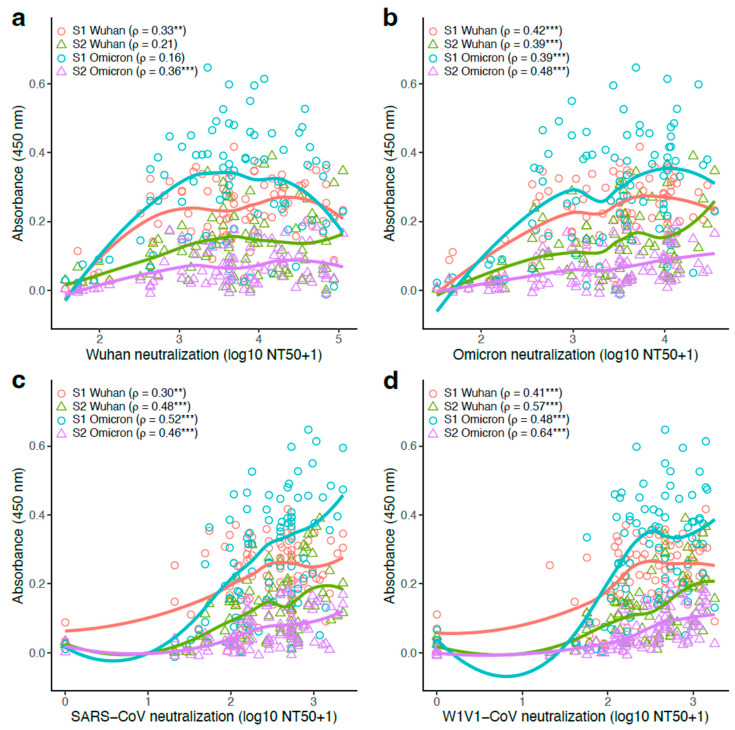
Antibody titers were predominantly positively correlated to pseudovirus neutralization. Binding (measured by ELISA) and neutralizing (measured as 50% neutralization titer; log10 NT50+1 transformation) antibody levels were compared for the 87 study participants. S1-Wuhan-, S2-Wuhan-, S1-Omicron-, and S2-Omicron-specific antibody levels vs. (**a**) Wuhan pseudovirus neutralization, (**b**) Omicron pseudovirus neutralization, (**c**) SARS-CoV pseudovirus neutralization, and (**d**) WIV1-CoV pseudovirus neutralization. Curves show Loess smoothing as a way to visualize patterns, but the data were analyzed using the Spearman’s rank correlation test with a Benjamini–Hochberg correction for multiple comparisons. Significance was defined as ** *Q* < 0.01, and *** *Q* < 0.001.

There was a positive correlation between neutralization capacity and antibody titers, with all 16 analyses resulting in a positive rank correlation coefficient (Figure 4). Furthermore, 14 of the 16 analyses had significantly positive coefficients (Spearman’s rank correlation; adjusted *p* < 0.05). Antibody correlations with the Wuhan pseudovirus were the weakest, with rank correlation coefficients between 0.16 and 0.36 (Figure 4a). For the remaining pseudoviruses, significant positive rank correlation coefficients were recovered in all analyses (Figure 4b–d). The highest correlation coefficients were found in analyses with WIV1-CoV, with ρ = 0.64 for S2-Omicron and ρ = 0.57 for S2-Wuhan (both adjusted *p* < 0.001; Figure 4d).

Although S1-specific antibody levels were generally higher compared with their S2-specific counterparts, the correlation between neutralization and antibody production was mostly stronger in S2- than in S1-specific analyses. For example, for the WIV1 pseudovirus, the rank correlation coefficient was higher for S2-Wuhan (ρ = 0.57) compared with S1-Wuhan (ρ = 0.41) as well as for S2-Omicron (ρ = 0.64) compared with S1-Omicron (ρ = 0.48) (Figure 4d). This pattern was found for five of the eight possible S1- vs. S2-specific comparisons.

### 3.7. Antibody Responses Observed with the Immunocompetent Participants Were Generally Higher than That of the Immunocompromised Participants

**Figure 5:**
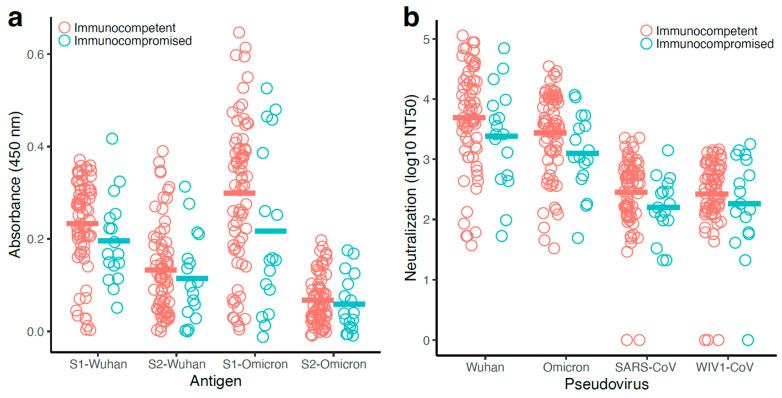
Lower, but not significant, binding and neutralization antibody levels were observed with the immunocompromised individuals. Binding and neutralizing antibody levels were compared for 70 immunocompetent and 17 immunocompromised study participants. NT50 values were transformed due to skew in the data (log10 of NT50+1 transformation). (**a**) S1- and S2-specific antibody levels measured by ELISA using S1-Wuhan, S2-Wuhan, S1-Omicron, and S2-Omicron as antigens. (**b**) Serum antibody neutralizing concentrations (50% neutralization titer; NT50) were determined against Wuhan, Omicron, SARS-CoV, and WIV1-CoV pseudoviruses. For each measurement, immunocompetent and immunocompromised means were analyzed using a *t*-test.

Since the POSITIVES cohort includes immunocompetent as well as immunocompromised participants, we evaluated the impact of immunocompromised status on coronavirus-specific humoral immunity. In this sub-study, 17 of the 87 samples were obtained from immunocompromised participants. Binding (Figure 5a) and neutralizing (Figure 5b) antibody titers were overall lower for the immunocompromised, although no comparison reached statistical significance; this may have been due to the lower number of individuals in the immunocompromised group, which was around 1/5 the size of the immunocompetent group.

### 3.8. Antibody Levels and Pseudovirus Neutralization Were Correlated More Strongly in Immunocompetent Participants

Rank-based correlations between antibody levels and pseudovirus neutralization titers were analyzed separately for immunocompromised and immunocompetent groups. In each case, S1- and S2-specific Wuhan and Omicron antibodies were tested with each pseudovirus in a separate analysis. For the immunocompromised group (Supplementary Figure S4), 15 of the 16 correlations were positive, but only three tests showed significant differences. For the WIV1-CoV pseudovirus, there was a significant positive correlation between neutralization and S2-Wuhan (Spearman’s rank correlation; ρ = 0.72; adjusted *p* < 0.01), S1-Omicron (Spearman’s rank correlation; ρ = 0.59; adjusted *p* < 0.01), and S2-Omicron (Spearman’s rank correlation; ρ = 0.68; adjusted *p* < 0.05) antibody levels.

Correlations between neutralization and antibody levels were considerably stronger in the immunocompetent group (Supplementary Figure S5), which had a larger sample size (n = 70) and thus greater statistical power compared to the immunocompromised (n = 17). Here, all 16 correlations were positive, of which 14 were significant. Analyses with the Wuhan pseudovirus had generally lower rank correlation coefficients, although there was a significant correlation for both Wuhan-S1 and -S2-specific antibodies and Omicron-S2-specific antibodies (Spearman’s rank correlation; adjusted *p* < 0.05; Supplementary Figure S5a). For Omicron, SARS-CoV, and WIV1-CoV pseudoviruses, significant positive rank correlation coefficients were found in all tests (Supplementary Figure S5c,d). The general pattern of stronger rank-based correlations between neutralization and antibody production for S2-specific antibodies, compared with S1-specific counterparts, was found among all participants as well as among immunocompetent participants alone.

### 3.9. Increased Number of Booster Doses Resulted in the Maintenance of High Antibody Levels After 2nd Booster

The impact of booster doses on the levels of antibody responses was examined separately for the immunocompromised and immunocompetent participants (Supplementary Figure S6). All the immunocompromised participants received at least one vaccine dose so group comparisons were limited to only vaccinated participants (Supplementary Figure S6a). Although higher S1- and S2-specific antibody levels were observed for three-, four-, and five-dose groups, the means of different dose groups were not significantly different from each other (linear mixed model with Tukey HSD test; *p* > 0.05; Supplementary Figure S6a). In analyses within each dose group of immunocompromised participants, significantly higher antibody levels were found in the four-dose S1- vs. S2-Omicron (paired *t*-test; *p* < 0.05), the five-dose S1- vs. S2-Wuhan (paired *t*-test; *p* < 0.05), and the five-dose S1- vs. S2-Omicron (paired *t*-test; *p* < 0.001) comparisons (Supplementary Figure S6a).

In the immunocompetent group, participants who had received multiple doses of vaccines had higher antibody levels than the non-vaccinated ones, while no significant difference was observed among the participants who had received various numbers of doses (doses 2–5; Supplementary Figure S6b). There were no immunocompetent participants who received only one dose, but those with two, three, four, and five doses all had higher mean antibody levels than the non-vaccinated immunocompetent participants (linear mixed model with Tukey HSD test; *p* < 0.001). There were no statistical differences in mean antibody levels among the two-, three-, four-, and five-dose groups (*p* > 0.05). Paired *t*-tests were used to evaluate if S1- and S2-specific antigens had equal means among the immunocompetent participants who received different numbers of doses. There were no significant differences in the antibody levels in the zero-dose group, but most comparisons in the other dose groups showed higher antibody levels against the S1-specific antigen, compared with its S2-specific counterpart (6 of 8 comparisons; *p* < 0.01). In general, booster doses did not result in high antibody levels but allowed for maintenance of moderate/high levels of antibodies after two doses, which may be critical for immunocompromised individuals.

We also examined the impact of booster doses on pseudovirus neutralization for the immunocompromised and immunocompetent participants and obtained similar results (Supplementary Figure S7). Mean neutralization levels among the dose groups did not differ significantly (linear mixed model with Tukey HSD test; *p* > 0.05; Supplementary Figure S7a). Neutralization was also similar across the different pseudoviruses for within dose group comparisons with three exceptions: Wuhan vs. SARS-CoV in the three-dose group, Wuhan vs. SARS-CoV in the five-dose group, and Wuhan vs. WIV1-CoV in the five-dose group (Supplementary Figure S7a).

An increased number of vaccine doses promoted a stronger response among the vaccinated participants within the immunocompetent group, compared with the unvaccinated ones (Supplementary Figure S7b). Those who had received two, three, four, and five doses had higher neutralization levels compared with non-vaccinated immunocompetent participants (linear mixed model with Tukey HSD test; *p* < 0.001). There were no statistical differences among mean neutralization levels in the two-, three-, four-, and five-dose groups (*p* > 0.05). Within each immunocompetent dose group, linear mixed models with patient ID as a random effect were used to compare Wuhan, Omicron, SARS-CoV, and WIV1-CoV pseudovirus neutralization levels for all within-dose group comparisons of vaccinated (2–5 doses) immunocompetent participants (linear mixed model with Tukey HSD test; *p* < 0.001; Supplementary Figure S7b). Overall booster doses did not generally result in higher neutralizing antibody levels, but the antibody levels were at least maintained after two doses, which may be critical for immunocompromised individuals.

## 4. Discussion

COVID-19-related health emergency restrictions were lifted across the globe by May 2023 [5,27]. However, SARS-CoV-2 infections continue to be recorded worldwide [6], which may be due to the ability of the virus to evade host immune responses, including neutralizing antibody-derived immunity. Most antibody escape mutations are associated with the S1 subunit of the spike protein, especially the RBD and the N-terminal domain (NTD) [14,15,16,18]. The other region of the spike, the S2 subunit, is the most conserved region amongst coronaviruses [29]. We hypothesized that S2-specific antibody responses are suboptimal in vaccinated and SARS-CoV-2-infected patients, resulting in an ineffective neutralization of distant coronaviruses.

Homology between the spike proteins of wild-type Wuhan, the distant SARS coronavirus (SARS-CoV), and the coronavirus of bat origin (WIV1-CoV) has been previously reported at 75.6% and 76.5% [28], respectively. Booster doses induced efficient neutralization of the wild-type Wuhan and the Omicron variants [28]. Our data were in accordance with these findings; booster immunizations provided higher neutralization potency against Wuhan and the Omicron variant, while the distant relatives WIV1-CoV and SARS-CoV were less sensitive to serum antibodies obtained from patients who had received Wuhan-trimer-based immunogens [28]. This weak neutralization of distant coronaviruses was observed despite the S2 region of Wuhan and Omicron sharing an almost 90% identity with SARS-CoV and WIV1-CoV.

We correctly predicted that S2-specific antibody levels are suboptimal in vaccinated COVID-19 patients, as confirmed by the lower S2-specific antibody levels in comparison with S1-specific antibody levels. Furthermore, these participants’ levels of Omicron-S1-antibody were higher than Wuhan-S1-antibody levels, but this difference did not translate into higher neutralization of Omicron pseudovirus in comparison with Wuhan pseudovirus. These data suggest that the infecting variants that originated from the Omicron variant may have had more impact on the potency of S1-specific antibodies. Omicron pseudovirus neutralization titers were lower despite the samples having higher Omicron-S1-specific antibody levels (in comparison with Wuhan-S1-specific antibody levels). However, Wuhan-S2-specific antibody levels were higher than Omicron-S2-specific antibody levels, which resulted in higher neutralization of Wuhan pseudovirus versus Omicron, suggesting a strong contribution of S2-specific antibodies to Wuhan/Omicron pseudovirus neutralization.

Furthermore, S1-specific antibody responses are not ideal for the prevention of future infection due to more frequent mutations in the S1-region [10,14,16,18]. Our data support the necessity of updating COVID-19 vaccine immunogens in favor of S2-only immunogens to potentially reduce S1-region dominance. In our study, correlations between neutralization and antibody levels were generally stronger for S2-specific antibodies, compared with S1-specific responses. We believe that vaccination outcome could have been improved with the use of SARS-CoV2 variant-derived spike immunogens. Nonetheless, we also believe that S2-specific antibody levels would have remained lower than S1 (variant strain), so cross-neutralization potency would have largely been unchanged.

The utilization of S2-specific immunogens for inducing cross-neutralizing antibody responses has been previously proposed [29,30,31,32]. This hypothesis is supported by the identification of cross-reactive neutralizing antibodies with potent neutralization activities against alpha CoVs, delta CoVs and some beta CoVs, and gamma CoVs [33,34,35,36,37]. For instance, mice were successfully immunized with an engineered S2-based immunogen, resulting in the development of neutralizing antibodies against multiple SARS-CoV-2 variants but also SARS-CoV, MERS-CoV, HCoV-229E, HCoV-NL63, and MjHKU4r-CoV-1 [38]. More recently, cross-neutralization antibodies were successfully induced following immunization with multivalent S2-tageting immunogens in transgenic K18hACE2 mice [39]. K18hACE2 mice were successfully protected against Omicron subvariant XBB as well as several sarbecoviruses with pandemic potential such as the bat sarbecovirus WIV1, BANAL-236, and a pangolin sarbecovirus [39]. Our data are in accordance with these findings, as greater correlations were associated with S2-specific antibody levels for neutralization of Wuhan, Omicron, SARS-CoV, and WIV1-CoV in comparison with S1-specific antibody levels. Similarly, the benefit of the avoidance of highly mutagenic regions in vaccine immunogens has been established with Stem-only immunogens against influenza virus [40].

We also evaluated the benefit of booster immunization on antibody responses. Although we found elevated levels of spike-specific antibody responses in immunocompromised individuals, these levels were lower than those observed in immunocompetent individuals. The presence of significantly lower neutralizing antibody titers for immunocompromised patients has been previously reported [16]. A contrasting result was reported by a study that found similar levels of S1-specific antibodies in 584 immunocompromised patients with hematologic cancers who had received a third COVID-19 mRNA vaccine booster and in their immunocompetent counterparts [41]. Additionally, the benefit of a third vaccine booster was highlighted in another study, which found elevated humoral immune responses in immunocompromised children who had earlier received a second booster vaccine [42]. More recently, a cohort study of 56 immunocompromised participants and 184 non-immunocompromised participants found that immunocompromised individuals had diminished SARS-CoV-2-specific humoral responses compared with the immunocompetent participants [10]. Our data are in accordance with these later findings. We found that neutralization antibody potency was lower in immunocompromised individuals than in the immunocompetent ones, but overall booster doses allowed for the maintenance of moderate/high levels of antibody responses. Despite the small sample size of the immunocompromised group, S2-specific antibodies appeared to better correlate with pseudovirus neutralization. Our findings confirm the benefit of vaccination against SARS-CoV-2, especially for immunocompromised individuals, because the presence of effector immune molecules, such as antibodies, can prevent transmission or at least reduce disease severity after exposure.

A limitation of this study is the potential bias with different starting dilutions of the plasma (10×, 20×, and 30×). The 96-well plate format did not provide enough space for serial dilution of plasmas with higher neutralization activities against Wuhan pseudovirus. A 384-well plate format would have been most appropriate, but we encountered challenges with consistency in our setting due to manual dilution of smaller volumes than those required for the 96-well plate format.

A major limitation to this study is the modest sample size, particularly within the immunocompromised group, which may explain the lack of statistical significance for some analyses. The study presented a bias in terms of female participation (80%) in comparison with the population of Massachusetts (51%). It is fair to say that vaccination constitutes an important tool to protect the population, as high levels of antibody responses were observed with a number of immunocompromised individuals. Similar benefits to vaccination were observed after a 2nd dose of COVID-19 immunization for cancer patients in a compilation of 21 studies, including a total of 5012 patients with cancer, of which 2676 (53%) had haematological malignancies, 2309 (46%) had solid cancers, and 739 were healthy controls [43]. Conversely, our current study also presented another subset of immunocompromised individuals with poor antibody responses. Consequently, it is advisable to measure levels of antibody responses following COVID-19 vaccination in order to identify those with weak antibody responses amongst immunocompromised individuals. Additional measures could then be readied for those with weak antibody responses to preemptively protect against disease severity. Another limitation, of this study is the lack of vaccinated but uninfected controls, which makes it difficult to distinguish between vaccine- or infection-induced antibody responses. Nonetheless, the higher levels of S1-omicron binding antibody responses suggest a more important role of infection (Omicron-derived) versus vaccination (Wuhan-based) for SARS-CoV-2-specific antibody production.

## 5. Conclusions

We found that the levels of S1-specific antibodies were higher than the levels of S2-specific antibodies in SARS-CoV-2-infected patients who had received a COVID-19 vaccine. However, S1-specific antibody responses are not the ideal target for the prevention of future infections by novel SARS-CoV-2 variants due to the potential for mutations outside of the S2 subunit. Our data confirm the validity of S2-based immunogens and support the use of S2-only immunogens to potentially reduce S1-region dominance. The lower levels of antibody responses observed with the immunocompromised individuals corroborate the necessity of booster doses to maintain effective antibody levels. Furthermore, these data advocate for the development of S2-targeting mAbs as a treatment strategy to prevent severe disease observed in some immunocompromised individuals who do not produce efficacious levels of antibodies.

## Figures and Tables

**Table 1:** Characteristics of study participants.

	Total	0 Doses	1 Dose	2 Doses	3 Doses	4 Doses	5 Doses
Patients (n = 87)							
Female (n = 60), %	69	6.7	0	8.0	34	10	10
Male (n = 27), %	31	3.4	1.1	2.3	10	8.0	5.7
Manufacturer of the most recent dose, %							
Johnson & Johnson (n = 1), %	1.1	0	1.1	0	0	0	0
Pfizer (n = 40), %	46	0	0	6.9	24	6.9	8.0
Moderna (n = 38), %	44	0	0	3.4	21	11	8.0
None (n = 8), %	9.2	NA	NA	NA	NA	NA	NA
Race %							
White (n = 69), %	79	6.9	1.1	6.9	36	13.8	13.8
Asian (n = 2), %	2.3	0	0	0	2.3	0	0
Black or African American (n = 6), %	6.9	0	0	1.1	2.3	2.3	1.1
Others and unknown (n = 10), %	11	2.3	0	2.3	3.4	2.3	1.1

## Data Availability

Data presented in this study are available by request to the corresponding author.

## Supplementary Materials

The following supporting information can be downloaded at: https://www.mdpi.com/article/10.3390/vaccines13090949/s1, Table S1: Number of participants, vaccine doses, days since first PCR/last vaccination/symptom onset, variant identity and Immunosuppression status; Table S2: Distance between the spike protein of 4 coronavirus; Table S3: Distance between the S1 proteins of 4 coronavirus and the 2 modified S1 protein; Table S4: Distance between the S2 proteins of 4 coronavirus and the 2 modified S2 protein; Figure S1: Soluble S1 and S2 antigens cluster with their natural occurring S1 and S2 counterparts; Figure S2: Pseudovirus neutralization titers were higher with Wuhan and Omicron variants compared to SARS-CoV and WIV1-CoV; Figure S3: Pseudovirus neutralization titers were higher with Wuhan and Omicron variants compared to SARS-CoV and WIV1-CoV; Figure S4: Antibody levels were generally positively, but not significantly, correlated to pseudovirus neutralization in immunocompromised participants; Figure S5: Antibody titers were predominantly positively and significantly correlated to pseudovirus neutralization in immunocompetent participants; Figure S6: S1- and S2-specific binding antibody levels were elevated for the booster groups but no statistical significance among doses was observed within the immunocompromised patients; Figure S7: Booster immunization significantly improved serum neutralization titers for immunocompetent and mixed results for the immunocompromised.
