# Supplementary material for: Cross-Neutralization of Distant Coronaviruses Strongly Correlates with Spike S2-Specific Antibodies from Immunocompetent and Immunocompromised Vaccinated SARS-CoV-2-Infected Patients
Source: Vaccines (Basel). 2025 Sep 4;13(9):949. doi: 10.3390/vaccines13090949 (PMC12474146; doi:10.3390/vaccines13090949)
Supplement: Supplementary file 1 [file vaccines-13-00949-s001.zip › vaccines-3808314-supplementary.pdf]

Supplementary Table S1: Number of participants, vaccine doses, days since first PCR/last vaccination/symptom onset, variant identity and Immunosuppression status.

	Number of vaccination doses	Immuno-suppression status	Number of days since last vaccination [range; mean]	Variant confirmation	Whole genome sequencing lineage	Number of days from first PCR [range; mean]	Latest vaccination (Number of days: n)	Number of days since symptom onset [range; mean]
Number of Participants (n=87)								
n=8	0	No (n=8) Yes (n=0)		Omicron (n=7); Un-sequenced (n=1)	BA.1.1 (n=2); BA1.1.16 (n=3) BA.2 (n=2)	[17; 15-22]		[19; 15-20]
n=1	1	No (n=0) Yes (n=1)	262	Omicron (n=1)	BA1.1.16 (n=1)	18	J&J (n=1)	20
n=9	2	No (n=8) Yes (n=1)	[393; 222-775]	Omicron (n=9)	BA.1 (n=5); BA1.1.16 (n=2); XBB.1.5 (n=1); XBB.1.16 (n=3)	[19; 14-29]	Pfizer (n=6); Moderna (n=3)	[20; 16-29]
n=39	3	No (n=34) Yes (n=5)	[201; 17-511]	Omicron (n=37); Un-sequenced (n=2)	BA.1 (n=3); BA1.1.16 (n=9); BA1.1 (n=3); BA.2 (n=9); BA.2.12.1	[22; 15-44]	Pfizer (n=21); Moderna (n=18)	[24; 12-70]

					(n=2); BA.5.2 (m=4); BA.5.5 (n=1) DS.1 (n=1) XBB.1.5.22 (n=1)			
n=16	4	No (n=10) Yes (n=6)	[149; 31-299]	Omicron (n=16)	BA2.12.1 (n=2); BA2 (n=1); BA.5.2 (n=3); BA.4.1 (n=1); BE.1.1.1 (n=1); BF.7 (n=1); XBB.1.5.21 (n=1); BQ.1.1 (n=1); BA.1 (n=1); XBB.1.5 (n=2)	[24; 16-48]	Pfizer (n=6) Moderna (n=10)	[24; 8-48]
n=14	5	No (n=10) Yes (n=4)	[135; 23-225]	Omicron (n=13); Un-sequenced (n=1)	BA.4.1 (n=1); BF.7.4 (n=1); BA.4.6.2 (n=1); XBB.1.5 (n=6); BF.7 (n=2); XBB.1.5.22 (n=1); FL4.6 (n=1)	[21; 16-34]	Pfizer (n=7) Moderna (n=7)	[23; 15-43]

Samples were collected between 14 days and 48 days from the first PCR test. SARS-CoV-2 variant information, Immunosuppression status and other clinical data were extracted from participant medical records.

Supplementary Table S2: Distance between the spike protein of 4 coronavirus

	Omicron	SARS-CoV	WIV1-CoV	Wuhan
**Omicron**		74.62%	75.18%	96.82%
**SARS-CoV**	74.62%		92.08%	75.61%
**WIV1-CoV**	75.18%	92.08%		76.41%
**Wuhan**	96.82%	75.62%	76.41%	

Supplementary Table S3: Distance between the S1 proteins of 4 coronavirus and the 2 modified S1 protein

	Omicron_S1	SARS-CoV_S1	Soluble_Omicron_S1	Soluble_Wuhan_S1	WIV1-CoV_S1	Wuhan_S1
**Omicron_S1**		63.25%	99.7%	94.94%	64.86%	95.05%
**SARS-CoV_S1**	63.25%		63.623%	64.93%	85.10%	64.53%
**Soluble_Omicron_S1**	99.70%	63.62%		95.24%	65.42%	95.24%
**Soluble_Wuhan_S1**	94.94%	64.92%	95.24%		66.87%	100%
**WIV1-CoV_S1**	64.86%	85.10%	65.42%	66.87%		66.28%
**Wuhan_S1**	95.05%	64.53%	95.24%	100%	66.28%	

Supplementary Table S4: Distance between the S2 proteins of 4 coronavirus and the 2 modified S2 protein

	Omicron_S2	SARS-CoV_S2	Soluble_Omicron_S2	Soluble_Wuhan_S2	WIV1-CoV_S2	Wuhan_S2
**Omicron_S2**		88.95%	98.86%	97.73%	88.77%	98.95%
**SARS-CoV_S2**	88.95%		87.69%	88.45%	99.47%	89.65%
**Soluble_Omicron_S2**	98.86%	87.69%		98.86%	87.5%	97.73%
**Soluble_Wuhan_S2**	97.73%	88.45%	98.86%		88.64%	98.86%
**WIV1-CoV_S2**	88.77%	99.47%	87.50%	88.64%		89.83%
**Wuhan_S2**	98.95%	89.65%	97.73%	98.86%	89.83%	

Supplementary Tables S2-S4: The amino-acid sequence analysis revealed high homology between the Wuhan spike and Omicron B1.1.529 spike proteins (96.82%, Supplementary Table S1) but lesser similarities between the Wuhan spike and SARS-CoV spike proteins (75.62%). The Wuhan spike and WIV1-CoV spike protein were also less similar (76.41%, Supplementary Table S1). S1 was the region most dissimilar between Wuhan and SARS-CoV proteins, and between Wuhan and WIV1-CoV (Supplementary Table S2). Wuhan S1 shared 95.73% identity with Omicron S1 but just 63.10% and 64.71% with SARS-CoV and WIV1-CoV, respectively (**Supplementary Table S2**). By contrast, the S2 sequences were highly homologous with Wuhan S2 and Omicron S2 sharing 98.95 % identity, Wuhan S2 and SARS-CoV S2 sharing 89.65% identity, and Wuhan and WIV1-CoV sharing 89.82% identity (**Supplementary Table S3**). Soluble S2 antigens were in fact highly similar to the non-modified protein sequences with soluble Wuhan S2 sharing 98.86% homology with naturally occurring Wuhan S2 and soluble Omicron S2 sharing 98.86% homology with naturally occurring Omicron B1.1,529 S2 (**Supplementary Table S3**).

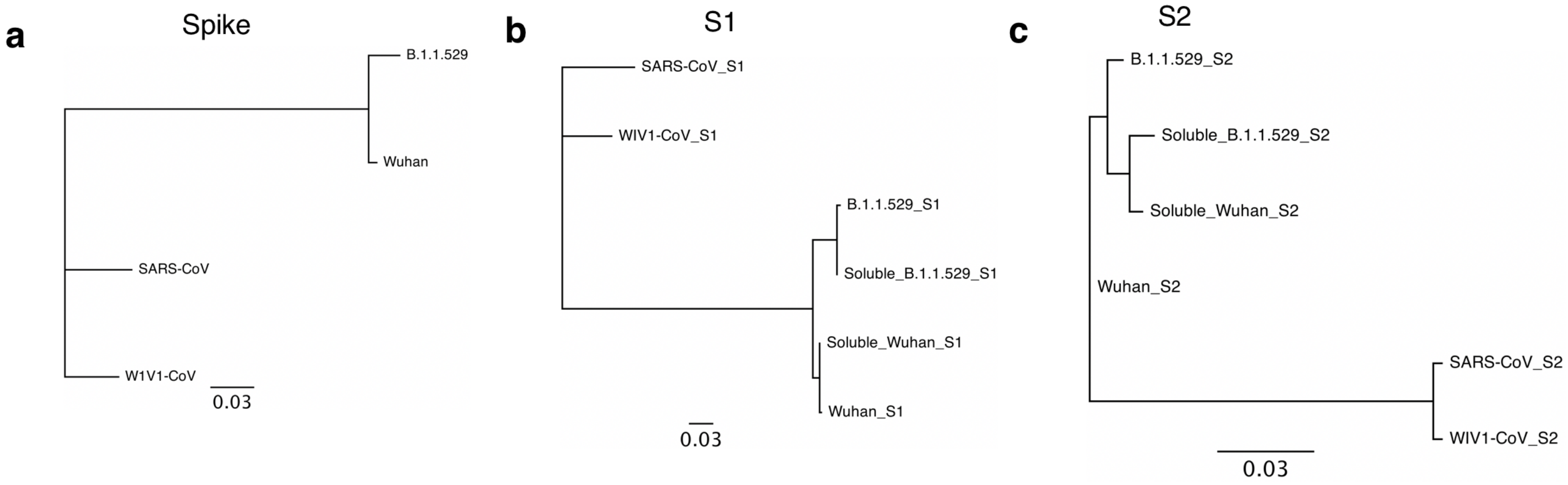

Supplementary Figure S1. **Soluble S1 and S2 antigens cluster with their natural occurring S1 and S2 counterparts.** Amino-acid sequence of Wuhan, Omicron (B1.1529), SARS coronavirus (SARS-CoV) and WIV1 (WIV1-CoV) coronavirus spike proteins were aligned using Geneious software. Phylogenetic trees were derived from the sequence alignment. (a) Comparison of the full-length spike protein sequences. The last 18 amino acids (Δ18) were excluded from the analysis to reflect the length of the proteins included in pseudovirus production. (b) Comparison of the full-length spike S1 subunits. (c) Comparison of the full-length spike S2 subunits. Soluble S1 and soluble S2 correspond to the sequence of the commercial antigen used for ELISA.

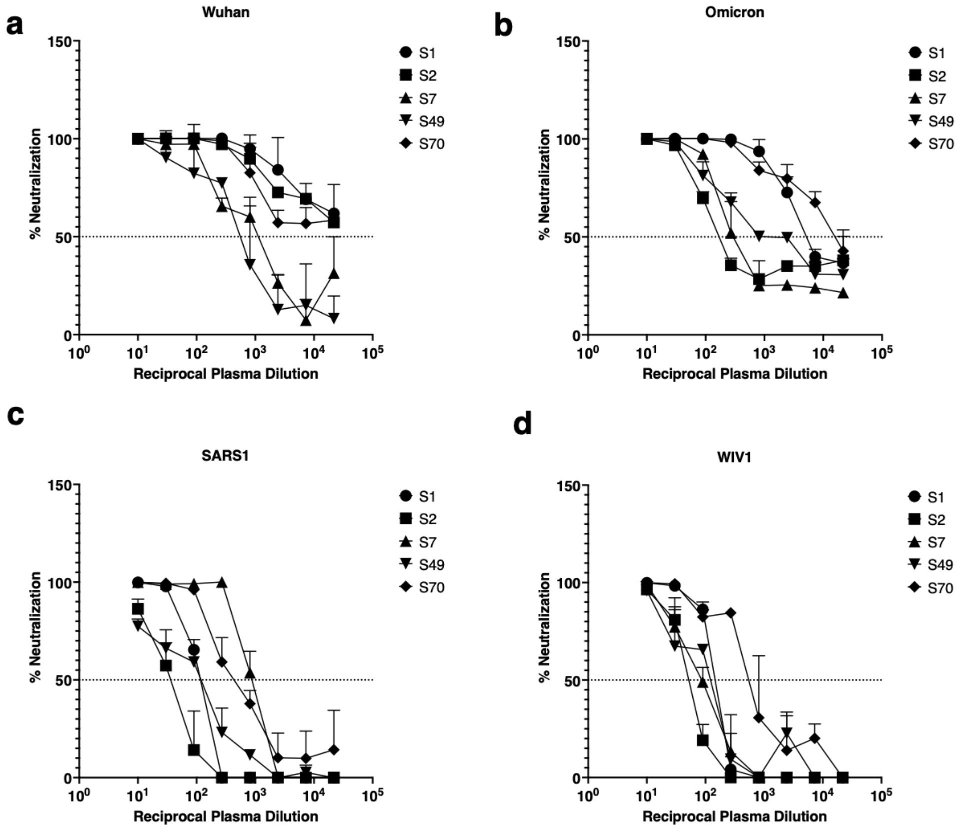

Supplementary Figure S2: Pseudovirus neutralization titers were higher with Wuhan and Omicron variants compared to SARS-CoV and WIV1-CoV

Serum neutralization concentrations were analyzed using GrapPad Prism software. Five representative samples (S1; S2; S7, S49 and S70) are presented (a) Wuhan, (b) Omicron, (c) SARS-CoV and (d) WIV1-CoV. Data were analyzed using GraphPad Prism software (version 9.5.0).

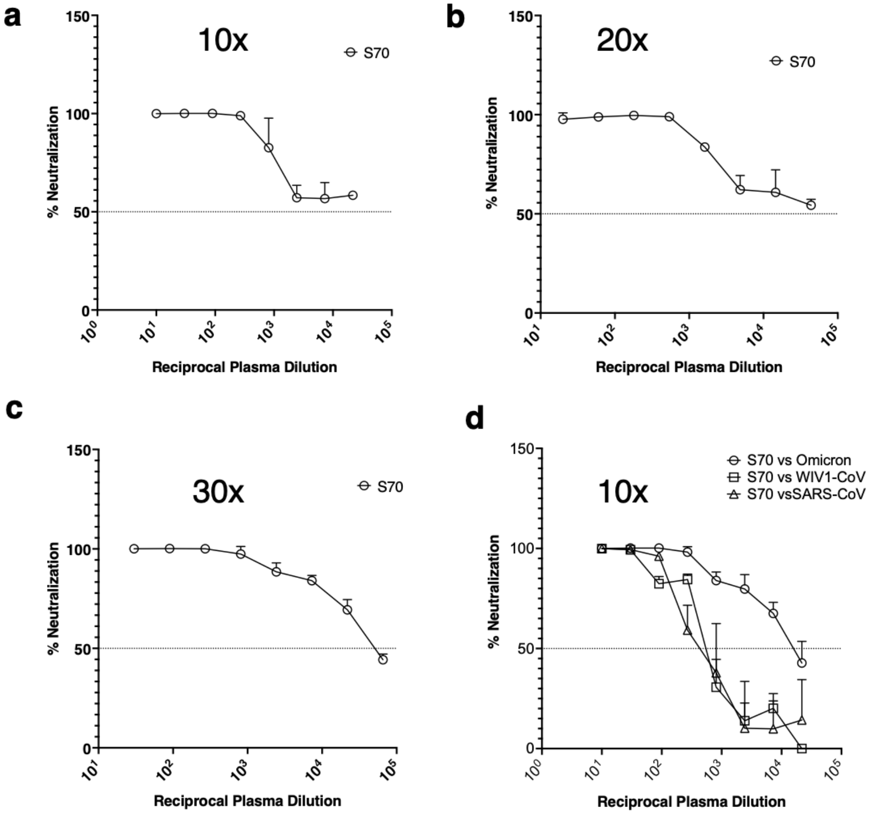

Supplementary Figure S3: Pseudovirus neutralization titers were higher with Wuhan and Omicron variants compared to SARS-CoV and WIV1-CoV

Serum neutralization concentrations were analyzed using GrapPad Prism software. Sample 70 (S70) strongly neutralized Wuhan pseudovirus at 10x starting dilution of plasma (a) and required additional starting dilutions (20x or 30x) for crossing the NT50% line. The same sample 70 strongly neutralized the Omicron but lower potencies were observed against SARS-CoV and WIV1-CoV (d) Representative Wuhan neutralization curves requiring additional starting dilutions (10x, 20x and 30x) Data were analyzed using GraphPad Prism software (version 9.5.0).

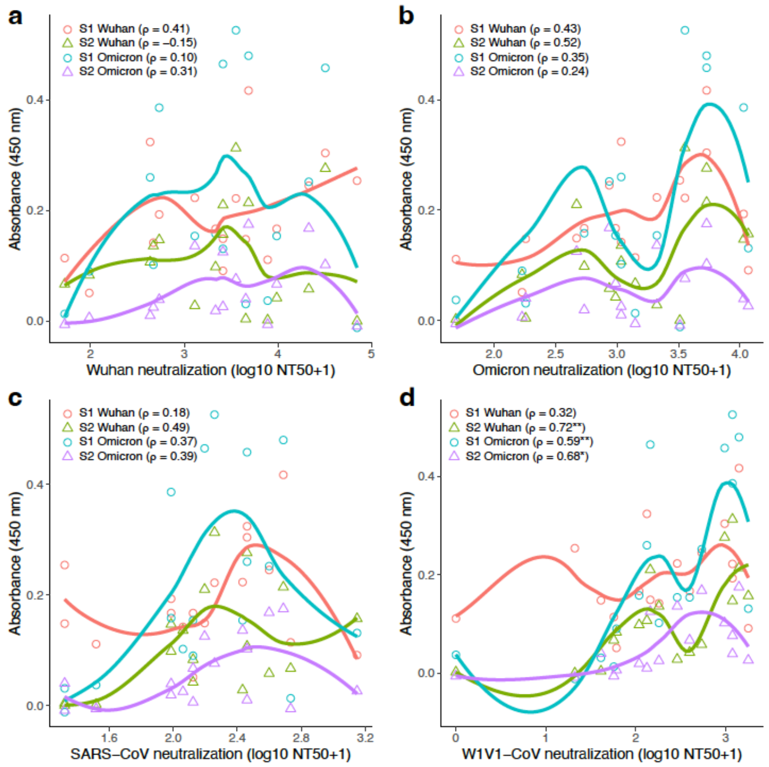

Supplementary Figure S4: Antibody levels were generally positively, but not significantly, correlated to pseudovirus neutralization in immunocompromised participants.

Binding (measured by ELISA) and neutralizing (measured as 50% neutralization titer; log10 NT50+1) antibody levels were compared for the 17 immunocompromised participants. S1-Wuhan, S2-Wuhan, S1-Omicron, and S2-Omicron specific levels vs (a) Wuhan pseudovirus neutralization, (b) Omicron pseudovirus neutralization, (c) SARS-CoV pseudovirus neutralization, and (d) WIV1-CoV pseudovirus neutralization. Curves show loess smoothing as a way to visualize patterns, but the data were statistically analyzed using the Spearman's rank correlation test with a Benjamini-Hochberg correction for multiple comparisons. Significance defined as *$Q < 0.05$, **$Q < 0.01$, and ***$Q < 0.001$.

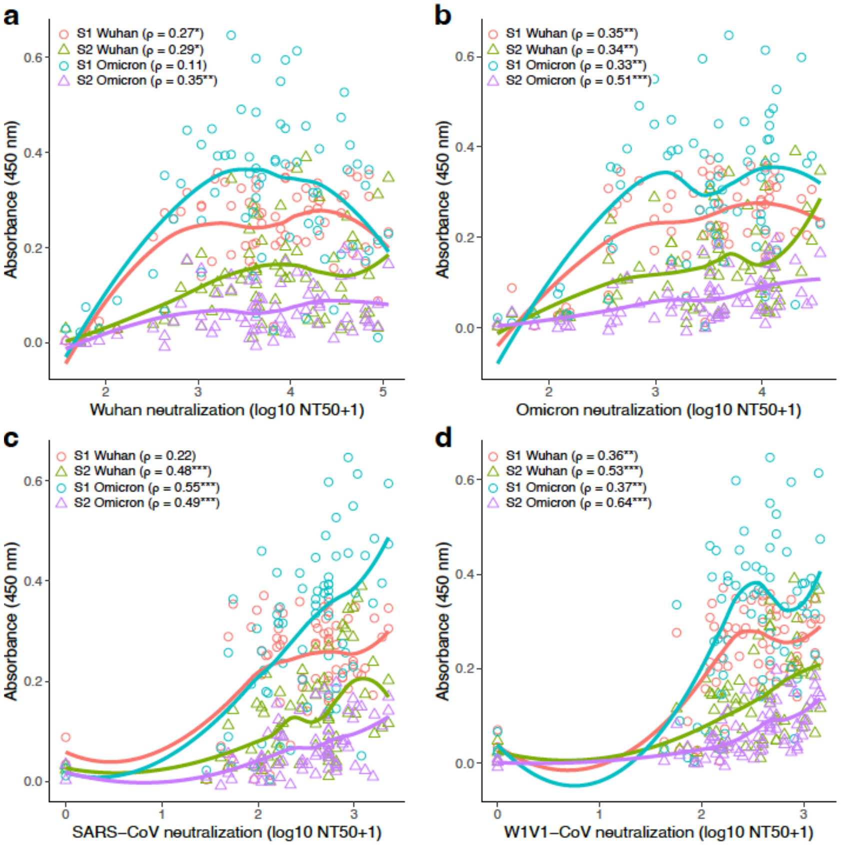

Supplementary Figure S5: Antibody titers were predominantly positively and significantly correlated to pseudovirus neutralization in immunocompetent participants.

Binding (measured by ELISA) and neutralizing (measured as 50% neutralization titer; log10 NT50+1) antibody levels were compared for the 70 immunocompetent participants. S1-Wuhan, S2-Wuhan, S1-Omicron, and S2-Omicron specific levels vs (a) Wuhan pseudovirus neutralization, (b) Omicron pseudovirus neutralization, (c) SARS-CoV pseudovirus neutralization, and (d) WIV1-CoV pseudovirus neutralization. Curves show loess smoothing as a way to visualize patterns, but the data were statistically analyzed using the Spearman's rank correlation test with a Benjamini-Hochberg correction for multiple comparisons. Significance defined as $*Q < 0.05$, $**Q < 0.01$, and $***Q < 0.001$.

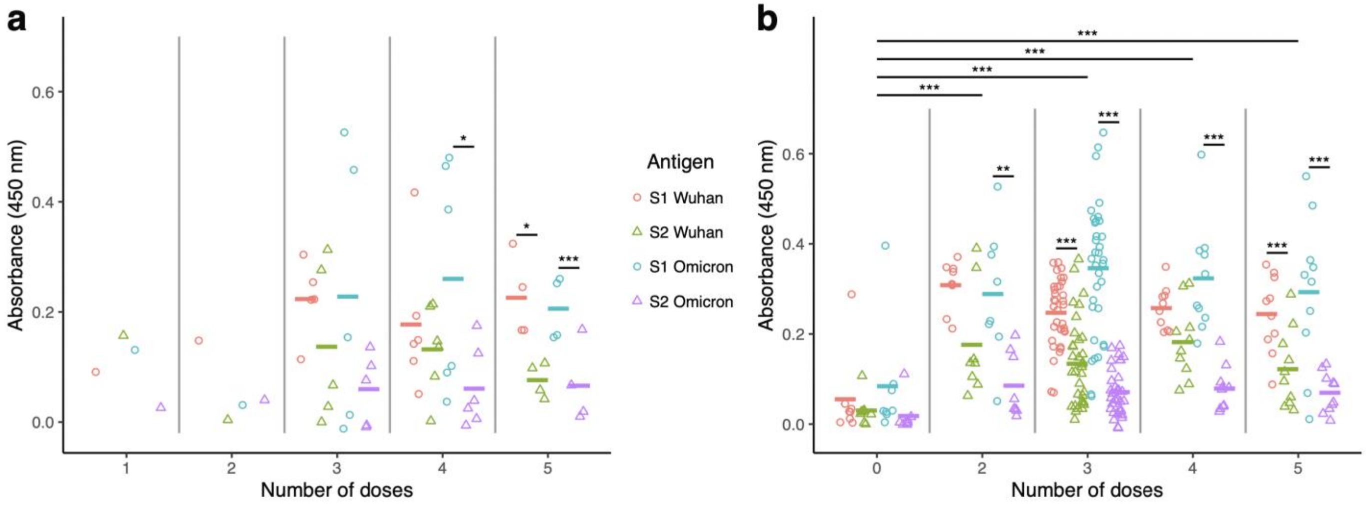

Supplementary Figure S6. S1- and S2-specific binding antibody levels were elevated for the booster groups but no statistical significance among doses was observed within the immunocompromised patients

Binding antibody levels were analyzed according to the number of doses for (a) immunocompromised and (b) immunocompetent participants. S1-Wuhan, S2-Wuhan, S1-Omicron and S2-Omicron-specific levels were determined by ELISA at OD 450nm. Dose means were compared with one-way ANOVA, and S1 vs S2 comparisons were done with paired *t*-tests. Statistical significance was defined as **P* < 0.05, ***P* < 0.01, and ****P*<0.001.

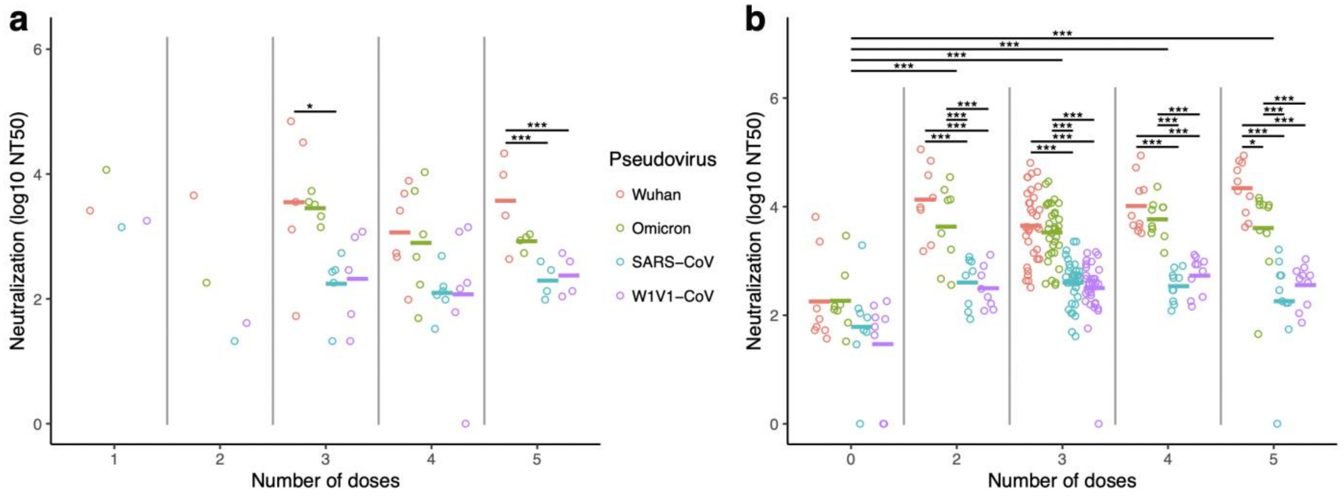

Supplementary Figure S7. Booster immunization significantly improved plasma neutralization titers for immunocompetent and mixed results for the immunocompromised.

Neutralizing antibody levels were analyzed according to the number of doses for (a) immunocompromised and (b) immunocompetent participants. Neutralization antibody concentrations were determined against Wuhan, Omicron, SARS-CoV and WIV1-CoV pseudoviruses. (a) Analysis with immunocompromised participants. (b) Analysis with immunocompetent participants. Dose means were compared with one-way ANOVA, and within dose numbers the pseudovirus NT50 values were compared with linear models and patient ID as a random effect. Statistical significance was defined as **P* < 0.05, ***P* < 0.01, and ****P*<0.001.
